# Na^+^/H^+^ Exchangers and Intracellular pH in Perinatal Brain Injury

**DOI:** 10.1007/s12975-013-0322-x

**Published:** 2014-01-24

**Authors:** Cristina Uria-Avellanal, Nicola J. Robertson

**Affiliations:** Neonatology, Institute for Women’s Health, University College London, 74 Huntley Street, 4th floor, Room 401, London, WC1E 6AU UK

**Keywords:** Brain pH_i_, NHE blockade, Neonatal encephalopathy, Brain alkalosis, Birth asphxyia, Neuroprotection, Hypoxia–ischemia

## Abstract

Encephalopathy consequent on perinatal hypoxia–ischemia occurs in 1–3 per 1,000 term births in the UK and frequently leads to serious and tragic consequences that devastate lives and families, with huge financial burdens for society. Although the recent introduction of cooling represents a significant advance, only 40 % survive with normal neurodevelopmental function. There is thus a significant unmet need for novel, safe, and effective therapies to optimize brain protection following brain injury around birth. The Na^+^/H^+^ exchanger (NHE) is a membrane protein present in many mammalian cell types. It is involved in regulating intracellular pH and cell volume. NHE1 is the most abundant isoform in the central nervous system and plays a role in cerebral damage after hypoxia–ischemia. Excessive NHE activation during hypoxia–ischemia leads to intracellular Na^+^ overload, which subsequently promotes Ca^2+^ entry via reversal of the Na^+^/Ca^2+^ exchanger. Increased cytosolic Ca^2+^ then triggers the neurotoxic cascade. Activation of NHE also leads to rapid normalization of pH_i_ and an alkaline shift in pH_i_. This rapid recovery of brain intracellular pH has been termed pH paradox as, rather than causing cells to recover, this rapid return to normal and overshoot to alkaline values is deleterious to cell survival. Brain pH_i_ changes are closely involved in the control of cell death after injury: an alkalosis enhances excitability while a mild acidosis has the opposite effect. We have observed a brain alkalosis in 78 babies with neonatal encephalopathy serially studied using phosphorus-31 magnetic resonance spectroscopy during the first year after birth (151 studies throughout the year including 56 studies of 50 infants during the first 2 weeks after birth). An alkaline brain pH_i_ was associated with severely impaired outcome; the degree of brain alkalosis was related to the severity of brain injury on MRI and brain lactate concentration; and a persistence of an alkaline brain pH_i_ was associated with cerebral atrophy on MRI. Experimental animal models of hypoxia–ischemia show that NHE inhibitors are neuroprotective. Here, we review the published data on brain pH_i_ in neonatal encephalopathy and the experimental studies of NHE inhibition and neuroprotection following hypoxia–ischemia.

## Introduction

Encephalopathy consequent on perinatal hypoxia–ischemia occurs in 1–3 per 1,000 term births in the UK and frequently leads to serious and tragic consequences that devastate lives and families, with huge financial burdens for society [1]. Although the recent introduction of cooling represents a significant advance, only around 40 % survive with normal neurodevelopmental function [2]. There is thus a significant unmet need for novel, safe, and effective therapies to optimize brain protection following brain injury around birth.

In this review, we discuss both preclinical and clinical research studies that have led to a better understanding of the pathophysiology and metabolic pathways following an acute hypoxic–ischemic perinatal event. Magnetic resonance spectroscopy (MRS) has been an important tool to understand the evolution of energy failure and brain intracellular pH (pH_i_) after hypoxia–ischemia [3]. Phosphorus-31 (^31^P) MRS has demonstrated an alkaline shift in brain pH_i_ [4]; those infants with worse cerebral injury on magnetic resonance imaging (MRI) have a more alkaline brain pH_i_ on ^31^P MRS [5]. It is likely that the rapid shift in brain pH_i_ to alkaline values following hypoxia–ischemia plays an important role in cell death.

The Na^+^/H^+^ exchangers (NHE) are a family of ion membrane transport proteins involved in maintaining a normal pH_i_ and cell volume in many mammalian cell types, by extruding protons in exchange for sodium influx into cells in an electroneutral manner. Excessive activation of NHE is likely to lead to the alkaline shifts in pH_i_ and increased cell death via Na^+^ overload, which promote intracellular Ca^2+^ entry. Here, we also review the role of NHE in hypoxia–ischemia, the evidence of neuroprotection with NHE blockade in animal models, and the important association of alkaline brain pH_i_ and seizures.

## Acute Hypoxic–Ischemic Perinatal Brain Injury in the Term Infant

Perinatal hypoxic–ischemic brain injury in the term baby is a significant problem throughout the world. Hypoxia–ischemia may occur acutely or chronically and is most commonly associated with maternal factors (hypotension, severe hypoxia), cord factors (prolapse, occlusion), placental factors (insufficiency and abruption), and uterine factors (rupture). Neonatal postnatal events such as shock, respiratory, or cardiac arrest can also lead to hypoxic–ischemic injury. Neonatal encephalopathy (NE) is the clinical manifestation of the ensuing disordered brain function and occurs in 1–3 per 1,000 live term births in the UK and other developed world countries [1]. The encephalopathy typically presents as respiratory difficulties, depression of tone and reflexes, subnormal level of consciousness, and often seizures. Serious consequences follow moderate to severe NE; these include death in 10–15 %, cerebral palsy in 15 %, and other significant cognitive, developmental, and behavioral problems in 40 % of survivors [6]. The financial and human costs to the family and society are thus very high.

Following two decades of laboratory studies [7, 8], clinical trials [2], and recent endorsement from regulatory bodies [9], therapeutic hypothermia is now the standard clinical care for moderate to severe NE in the UK and developed world. Meta-analysis [2, 10] of three large pragmatic trials [2, 11–13] show that therapeutic hypothermia reduces death or disability at 18 months with a risk ratio of 0.81 (95 % CI 0.71–0.93) and a number needed to treat of 6–7. Importantly, data are emerging, which confirm that the favorable outcome of cooled babies at age 18 months is associated with favorable outcome at age 7–8 years [14].

However, hypothermia is only partially effective after neonatal hypoxia–ischemia, and 40 % of all treated infants still suffer severe neurodevelopmental disability or death despite treatment. Additional neuroprotective agents are needed either to augment hypothermic neuroprotection [15] or to use alone in settings where hypothermia has not been established as standard care.

### Pattern of Brain Injury on Conventional MRI

Understanding the fundamental mechanisms underlying brain injury in the newborn brain at term has rapidly advanced in recent years. Using MRI, the patterns of hypoxic–ischemic brain injury that have been described in pathology studies in the past can now be demonstrated in superb detail and resolution in vivo in human neonates [16, 17]. Using MRS, the timing of the evolution of energy failure and changes in brain pH_i_ have been fundamental in suggesting new avenues for neuroprotection.

**Fig. 1:**
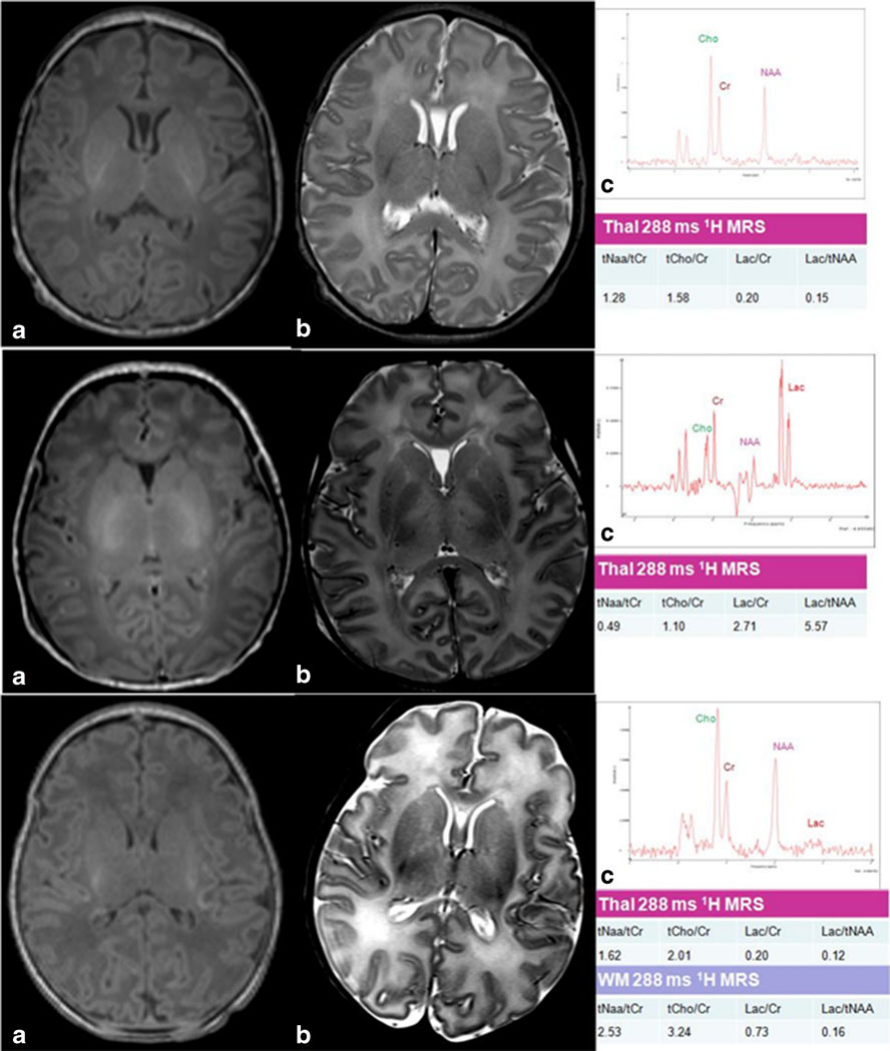
Patterns of brain injury on MRI. *Top row* (3 Tesla): **a** axial T1-weighted (*w*) MPRAGE and **b** T-2 w 2D 3 mm 3 T MRI of a term infant with normal intracranial appearances on day 5 after birth. This infant presented with stage 1 (*mild*) neonatal encephalopathy. A normal signal intensity is seen from the posterior limb of the internal capsule on **a** and **b**. A thalamic proton (^1^H) magnetic resonance (MR) spectrum TE 288 ms from the same infant is shown in **c**. The lactate peak at 1.3 ppm is just visible. *Middle row* (3 Tesla): **a** axial T1-w MPRAGE and **b** axial T2-w 2D 3 mm of an infant with a predominant BGT pattern of brain injury (involvement of basal ganglia, thalami, and perirolandic cortices). A thalamic ^1^H MR spectrum TE 288 ms from the same infant is shown in **c**. A high thalamic Lac/NAA ratio of 5.57 is observed. *Bottom row* (1.5 Tesla): **a** axial T1-w; **b** axial T2-w 2D 3 mm of an infant with neonatal encephalopathy with a predominant watershed injury pattern. Areas of cerebral cortical infarction affecting the insular cortices, frontal, and temporal occipital lobes in anterior and posterior arterial watershed territory is seen. A thalamic ^1^H MR spectrum TE 288 ms from the same infant is shown in **c**. The Lac/NAA is normal in the thalamus. The white matter spectrum (not shown) has a raised Lac/Cr peak area ratio

The two main patterns of injury on MRI have been clearly described in the 1950s–1970s in a primate model: the injury pattern associated with acute total asphyxia [18] and with chronic partial asphyxia [19, 20]. These two main patterns are described below and in Fig. 1. A normal neonatal MR image is shown in Fig. 1 (top row). The corresponding ^1^H MR spectra acquired from the left thalamus are shown for each set of MR images. The ^1^H MRS values from the white matter are also shown for the watershed predominant pattern of brain injury.

*Predominant basal ganglia–thalamus pattern (BGT)* is the most frequent pattern in “acute asphyxia,” affecting bilaterally the central grey nuclei (ventrolateral thalami and posterior putamina) and perirolandic cortex (Fig. 1 middle row). The hippocampus and brainstem can also be involved. This pattern of injury is most often seen following an acute sentinel event, e.g., uterine rupture, placental abruption, or prolapsed cord [21]. The basal ganglia and thalami are susceptible to acute perinatal hypoxic–ischemic injury due to their high metabolic rate and increased concentration of NMDA receptors [22]. Survivors face a range of functional impairments, which include cerebral palsy, feeding problems, speech and language problems, visual and hearing impairment, later seizures, behavioral difficulties, and cognitive impairment. Cerebral palsy affects three quarters of infants with NE with BGT lesions, and the severity of the BGT lesions is the best predictor of motor problems. A normal signal intensity of the posterior limb of the internal capsule within the neonatal period following neonatal encephalopathy is a good predictor of the ability to walk at 2 years [23].

*Watershed predominant pattern of injury* typically follows “prolonged partial asphyxia.” The areas involved are the vascular watershed zones (anterior–middle cerebral artery and posterior–middle cerebral artery), affecting white matter and in more severely affected infants the overlying cortex (Fig. 1, bottom row). The lesions can be uni- or bilateral, posterior and/or anterior. Conventional MRI can detect the loss of the cortical ribbon [24, 25]. These lesions may become cystic, atrophy, or develop gliosis [26]. Common associated etiological factors are hypotension, infection, and hypoglycemia [27]. Infants with a watershed pattern of injury have predominantly cognitive impairments often without functional motor deficits. Cognitive deficits include memory impairments, visual–motor or visual–perceptive dysfunction, or increased hyperactivity, sometimes in the absence of functional motor problems [28–32].

### Cerebral Energy Metabolism Following Perinatal Hypoxia–Ischemia

**Fig. 2:**
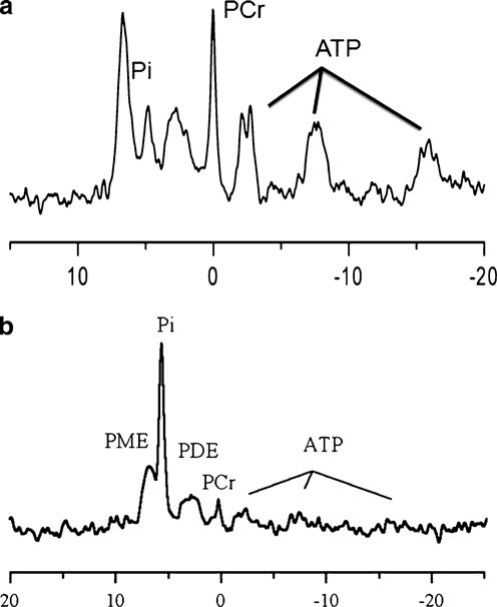
Representative ^31^P MRS spectra from **a** a normal baby and **b** a baby with severe neonatal encephalopathy. Seven main peaks can be assigned in a cerebral ^31^P MR spectrum: phosphomonoesters (*PME*), inorganic phosphate (*Pi*), phosphocreatine (*PCr*), phosphodiesters (*PDE*), and the three phosphate groups (α, β, and γ) in nucleotide triphosphate (*NTP*). The chemical shift difference between PCr and Pi forms the basis of intracellular pH measurement in the in vivo brain

**Fig. 3:**
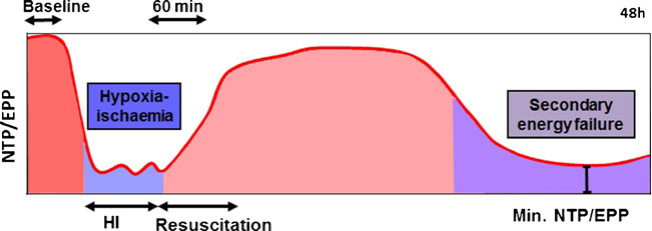
Schematic diagram illustrating the biphasic pattern of energy failure associated with a transient hypoxia–ischemic insult visualized using ^31^P MRS in the UCL piglet model. Nucleotide triphosphate (*NTP*)/exchangeable phosphate pool (EPP = Pi + PCr + NTP) is shown on the *y*-axis. The change in NTP/EPP during transient hypoxia–ischemia (HI), resuscitation, the latent phase (period between the recovery from acute HI and the evolution of secondary energy failure (SEF)), and SEF itself are shown. During the acute energy depletion, some cells undergo primary cell death, the magnitude of which will depend on the severity and duration of HI. Following perfusion, the initial hypoxia-induced cytotoxic edema and accumulation of excitatory amino acids typically resolve over 30–60 min with apparent recovery of cerebral oxidative metabolism (latent phase). It is thought that the neurotoxic cascade is largely inhibited during the latent phase and that this period provides a “therapeutic window” for therapies such as hypothermia and other agents. Cerebral oxidative metabolism may then secondarily deteriorate 6–15 h later (as SEF). This phase is marked by the onset of seizures, secondary cytotoxic edema, accumulation of cytokines, and mitochondrial failure

MRS has been used to study brain energy metabolism noninvasively; over the last 30 years phosphorus-31 (^31^P) and proton (^1^H) MRS have provided unique information on cerebral energy metabolism during the evolution of brain injury following hypoxia–ischemia in the newborn infant [33], neonatal rat [34], and the newborn piglet [3, 35] (Fig 2). In human infants, shortly after intrapartum hypoxia–ischemia ^31^P MRS often reveals normal cerebral energetics [36]. However, in infants with adverse outcome, despite adequate oxygenation and circulation, phosphocreatine (PCr) and nucleotide triphosphate (NTP, mainly ATP) decline, and Pi increases, in the first days of life [33, 36–38]. Brain pH_i_ has been observed to become alkaline during this phase [4, 5] (see next section). These metabolic changes were termed “secondary energy failure” (SEF) on the basis that they followed impaired intrapartum cerebral energy generation (resulting in transiently reduced PCr and NTP and increased Pi), which resolved following resuscitation [3] (Fig. 3). It was assumed that SEF was consequential to a pathological mechanism initiated by intrapartum hypoxia–ischemia or/and reperfusion/reoxygenation. ^1^H MRS provides complementary information to ^31^P MRS (in particular cerebral lactate, a marker of anaerobic metabolism and *N*-acetyl-aspartate (NAA), an abundant amino acid found mostly in neurons in the central nervous system (CNS) [39]). Because of the greater sensitivity of the ^1^H nucleus, data can be obtained from smaller regions of the brain. Cerebral lactate rises and NAA falls during transient hypoxia–ischemia; these metabolites return almost to baseline levels after successful resuscitation, only to be followed by a secondary increase in lactate and slower reduction in NAA in the hours that follow. These ^1^H MRS changes occur in parallel to the energy disruption (reduction in PCr/EPP and NTP/EPP) seen on ^31^P MRS. A recent meta-analysis of the prognostic accuracy of MR methods demonstrated that thalamic ^1^H MRS Lac/NAA peak area ratio acquired between days 5 and 14 after birth is a highly sensitive and specific biomarker of long-term neurodevelopmental outcome in infants with neonatal encephalopathy [40].

### Brain pH_i_ Changes in Babies with Neonatal Encephalopathy

^31^P MRS is the only noninvasive way to measure pH_i_ apart from positron emission tomography, which requires injection of radioactive ligands and is not feasible in babies. Using ^31^P MRS, brain pH_i_ can be measured using the chemical shift difference of Pi from PCr [41], or phosphoethanolamine from PCr [42, 43] or ATP from PCr [44]. The pH_i_ value calculated from the chemical shift difference of Pi form PCr is thought to reflect the pH_i_ in dead or injured cells, whereas that derived from other metabolites may reflect different cell populations. A summary of the studies of brain pH_i_ using ^31^P MRS performed in normal neonates and infants is shown in Table 1.

**Table 1:** Studies in normal neonates and infants documenting brain pHi using ^31^P MRS (based on [36, 38, 151–156])

Reference	*n*	GA at birth (weeks)	Age when studies	Mean brain pH_i_	Localization
Hope et al. [36]	6	Median 40 (28–40)	Mean 76 h (16 h–97 days)	7.14 ± 0.10	Surface coil
Hamilton et al. [151]	18	Median 32 (28–42)	5 (1–61) days GA + PNA median 35 weeks (28–42)	6.98 ± 0.34 (28 weeks)	Surface coil
Boesch et al. [152]	12	?	GA + PNA median 43 weeks (33 weeks–6 years)	7.08 (SD 0.1)	Surface coil
			8 were studied at 40 weeks		
Azzopardi et al. [153]	30 (data from 23)	Median 33 (24–42)	GA + PNA median 34 weeks (26–42)	7.1—no change with maturation	Surface coil
				AGA 28 weeks 7.14 (±0.28)	
				42 weeks 7.09 (±0.28)	
				SGA 28 weeks 6.97 (±0.24)	
				42 weeks 7.19 (±0.24)	
Laptook et al. 1989 [154]	7	40±1	10 examinations within the first 2 weeks after birth	7.02 ± 0.08	Surface coil
Van der Knapp et al. [155]	41	Term	Mean 71 months (1 months–16 years)	7.04 (95 % CI 6.96–7.12)	Volume localized (ISIS)
				No significant change	
Buchli et al. [156]	16^a^	Term	2–28 days GA + PNA mean 42 weeks (39–44)	7.11 (±0.06)	Volume localized (ISIS)
Martin et al. [38]	10^a^	Median 40 (36.3–42.1)	Median 4.3 days	7.12 (±0.05)	Volume localized (ISIS)
Robertson et al., unpublished data	3	Median 39 (38–40)	GA + PNA median 44 weeks (41–64)	7.02 (±0.03)	Volume localized (ISIS)

*GA* gestational age, *PNA* postnatal age

^a^Controls were healthy term newborn babies who had been hospitalised for non-neurologic reasons

**Fig. 4:**
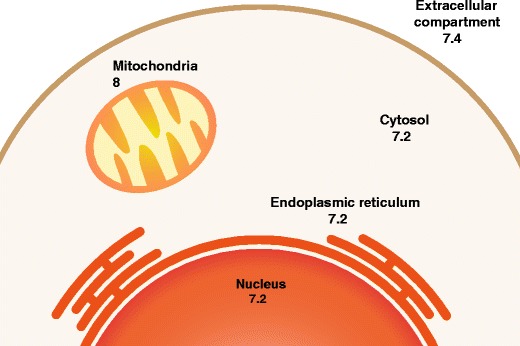
A schematic diagram showing the pH of the main cellular compartments in a typical mammalian cell (from Casey et al. [45]) The mitochondrial pH represents that in the inner mitochondrial membrane

**Fig. 5:**
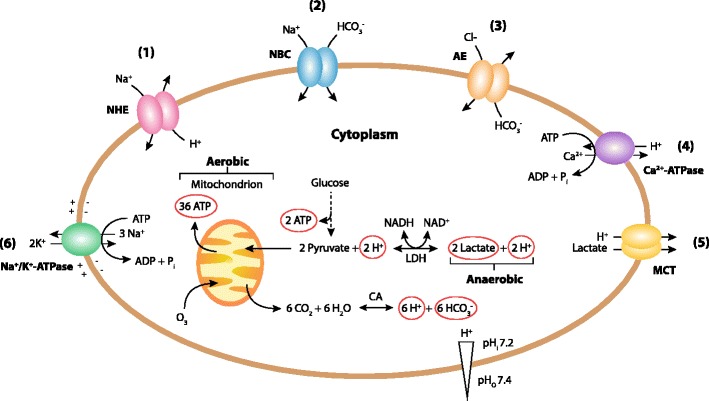
Ion transporters that regulate cytoplasmatic pH_i_. There is a tendency to acidification of the cytoplasm due to the activity of the anaerobic metabolism producing lactate from glucose and aerobic metabolism (oxidative phosphorylation in mitochondria that produce CO_2_). The main transporters regulating cytosolic pH are the plasma membrane Na^+^/H^+^ exchangers (NHEs) (see (*1*) on the diagram) and the Na^+^/HCO_3_^−^ cotransporters (NBCs) (*2*). The plasma membrane Cl^−^/HCO_3_^−^ or anion exchangers (*AE*) (*3*) counterbalance these mechanisms by acidifying the cell, and plasma membrane Ca^2+^-ATPases (*4*) also acidify the cytosol by exchanging cytosolic Ca^2+^ for extracellular H^+^ when intracellular Ca^2+^ is elevated. Importantly in the brain after hypoxia–ischemia, the monocarboxylate-H^+^ co-transporters (*MCTs*) will alkalinize the cell (*5*). The Na^+^/K^+^-ATPase pumps (*6*) establish an inward electrochemical Na^+^ gradient. Adapted from (from Casey et al. [45]). *CA* carbonic anhydrase, *LDH* lactate dehydrogenase, *pH*_*i*_ intracellular or cytosolic pH, *pH*_*o*_ extracellular or outside pH

Brain pH homeostasis in the cytosol and other cellular compartments is maintained by a dynamic, finely tuned balance between proton-extruding and proton-importing processes [45]. Under physiologic conditions, the extracellular pH is ∼7.4; however, the cytosolic pH is more acid (∼7.2). Other organelles possess their own specific pH (Fig. 4). Almost all proteins depend on pH to maintain their structure and function; pH plays an important part in many metabolic functions and the charge of biological surfaces affects many cellular reactions. There is a tendency for the cytosol to accumulate acid (from metabolic reactions—ATP production in the cytoplasm by glycolysis and in mitochondria by oxidative phosphorylation) (Fig. 5). Protons are actively extruded from the cytosol by proton pumping ATPases, coupling to other substrates through exchangers; the main transporter that protects cells against acidification is the Na^+^/H^+^ exchanger (see “Brain pH_i_ and Seizures”).

**Fig. 6:**
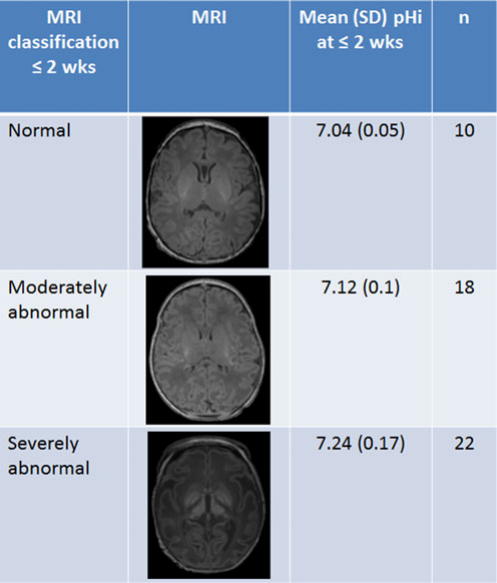
Association of the severity of brain injury in conventional MRI and mean brain pH_i_ within the first 2 weeks of age in 50 infants with mild, moderate, and severe neonatal encephalopathy. Mean (SD) brain pH_i_ at <2 weeks of age in infants with neonatal encephalopathy classified according to the brain MRI pattern (data from Robertson et al. [5]). There are significant differences between the brain pH_i_ in the group with a normal brain MRI and the brain pH_i_ in the group with a severely abnormal MRI

In 2002, we studied the chemical shift difference of Pi in whole-brain ^31^P MRS in relation to outcome in babies with neonatal encephalopathy [5]. In 78 babies with neonatal encephalopathy studied serially during the first year after birth (151 studies throughout the year including 56 studies of 50 infants during the first 2 weeks after birth), we demonstrated that: (1) alkaline brain pH_i_ was associated with severely impaired outcome in babies; (2) the degree of brain alkalosis was related to the severity of brain injury on MRI and brain lactate concentration (Fig. 6); and (3) brain alkalosis persisted for several weeks in babies with a severely impaired outcome and persistence was associated with cerebral atrophy on MRI. A brain pH_i_ of 7.15 and above measured in the first 2 weeks after birth had a sensitivity of 71 % and specificity of 92 % for predicting adverse outcome. Published studies that have measured brain pH_i_ using ^31^P MRS in infants with birth asphyxia are listed in Table 2.

**Table 2:** Studies in infants with birth asphyxia documenting brain pHi measured by ^31^P MRS (based on [4, 5, 33, 36, 38, 151, 154, 157])

Reference	*n*	GA at birth (weeks)	Age when studies	Mean brain pH_i_	Localization
Cady et al. [157]	7 (3 birth asphyxia)	33–40	42 h–26 days	7.2 (SD 0.1)^a^	Surface coil
				One infant studies on 5 occasions (on day 26: brain pH_i_ = 7.4)	
Hope et al. 1984 [36]	10	38–41	8 h–27 days (obtained in 3–6 times in each infant)	7.17 ± 0.1 at time of lowest PCr/Pi (mean age 113 h: 16 h–9 days)	Surface coil
Hamilton et al. [151]	27 Echodensities seen on USS (13 birth asphyxia)	Median 40 (27–42)	Median 3 days (8 h–23 days)	“Tended to be raised.” Values from 19 of the infants were above the regression line for normal infants; 6 were above the 95 % CI	Surface coil
Laptook et al. [154]	1	36	1.5, 8, 15 days, 9 months	7.3–7.4	Surface coil
	5	38 ± 2	Within first 2 weeks after birth	7.14 (±0.12) Significantly different from the normal infants when other ^31^P metabolites showed no difference	
Azzopardi et al. [33]	61 (all infants)	27–42	3 days (0–23)	7.14 ± 0.27 (*n* = 61)	Surface coil
	40 infants with asphyxia	40 (31–42)	3 days (1–10)	7.16 ± 0.19 (*n* = 36)	
Martin et al. [38]	23	Median 40 (36.9–41.9)	Median 3 days (NNS 1—severe)	7.21 (±0.16)	Volume localized (ISIS)
			Median 5.5 days (NNS 2—moderate)	7.09 (±0.04)	
			Median 3 days (NNS 3—mild)	7.10 (±0.08)	
Robertson et al. [4]	43	Median 39.5 (36–42)	77 examinations: 25 within 2 weeks age 16 between 2 and 4 weeks	At time of lowest PCr/Pi (within first 2 weeks of age)	Volume localized (CSI)
			25 between 4 and 30 weeks	7.23 (±0.07)—abnormal outcome group	
			11 when >30 weeks old	7.08 (±0.04)—normal outcome group	
Robertson et al. [5]	78	39.5 (SD 1.6) (36–42)	151 examinations within the first year after birth. 50 infants studies within 2 weeks of age	At time of lowest PCr/Pi (within first 2 weeks of age)	Volume localized (ISIS)
	35 (+ 43 presented above)			7.28 (±0.15)—23 infants with severe outcome or died	
				7.11 (±0.09)—normal outcome group	

*NNS* neonatal score, *NNS1* severe NE, *NNS 2* moderate NE, *NNS 3* mild NE, *GA* gestational age, *PNA* postnatal age

^a^The pH_i_ was taken from a heterogeneous group of infants including infants with congenital muscular dystrophy and with meningitis. The author noted there was no evidence of intracellular acidosis in infants with birth asphyxia and stated that the pH_i_ was similar in infants with birth asphyxia to those without. On close examination, however, infants with birth asphyxia tended to have a more alkaline pH_i_

In vivo data suggest that the return of pH_i_ to normal or alkaline values may be deleterious to cells that have undergone hypoxia–ischemia. This rebound alkalosis has been termed pH paradox and has been described in several cell types [46–48]. Possible mechanisms leading to pH-dependent injury include activation of phospholipases and proteases, which have an alkaline p*K*a, onset of the mitochondrial permeability transition pore, leading to uncoupling of oxidative phosphorylation and aggravation of ATP depletion [49], and an exacerbation of excitotoxic neuronal injury due to an increased NMDA activation at alkaline pH_i_ [50, 51]. An understanding of one of the main transporter groups leading to brain alkalosis after hypoxia–ischemia (the NHEs) is therefore important for future neuroprotection strategies.

## Na^+^/H^+^ Exchangers

The NHEs are a family of ion integrate membrane transport proteins involved in maintaining pH_i_ and cell volume in many mammalian cell types [52, 53], by extruding protons from, and taking up sodium ions into cells in an electroneutral manner (1:1 stoichiometry) (Fig. 5).

To date at least ten NHE isoforms (NHE1 to NHE10) have been identified in mammals [54]. NHE10 was recently described as an osteoclast-specific member of NHE family, regulating osteoclasts differentiation and survival [55]. NHE1–5 are expressed on the plasma membranes in various types. NHE6–9 reside on intracellular organellar membranes of the endosomal-trans Golgi network [56, 57]. NHE isoforms have similar membrane topologies, with an N-terminal membrane domain consisting of 12 predicted transmembrane segments and a more divergent C-terminal cytoplasmic domain [45, 57]. The NHE1 isoform has been studied closely and is present at the cell surface of most cells, especially plasma membranes. It is the most abundant isoform in the CNS [58, 59]. NHE1 plays a crucial role in protecting cells from internal acidification, acting together with bicarbonate-transporting systems and restoring cell volume to steady-state levels [60].

Intracellular acidosis is the major stimulus that regulates NHE1 activity. As the H^+^ concentration of the cytosol rises, a rapid increase in the activity of the transporter occurs. Near-maximal velocity is achieved in approximately one pH unit (Hill coefficient >1), thereby minimizing exposure of the cytoplasm to excess acidification [61, 62].

In addition to responding to intracellular acidification, various signals can alter the NHE1 internal pH sensitivity, such as hormones, mitogens, and physical stimuli (e.g., mechanical stretch and hyperosmolarity), modulating its state of phosphorylation [45, 53, 63–67]. The exchange activity is more active at alkaline pH values due to a shift of the set point by phosphorylation of the residues. It is also regulated at the transcriptional level, supporting the control at mRNA levels and at protein production level [68, 69]. An alkaline cytoplasmic pH (pH_c_) is thought to provide a permissive environment for the progression of diverse cellular processes, including changes in cell shape [70], adhesion [71], migration [72, 73], chemotaxis [74, 75], and proliferation [76–78].

### Effect of Hypoxia–Ischemia on NHE

Reductions in blood flow with ischemia and reduction in oxygen supply with hypoxia decrease the supply of oxygen required to maintain tissue ATP levels, especially in excitable organs such as the heart and brain that have a high demand for energy. As ATP stores are depleted, lactate, pyruvate, and protons accumulate owing to anaerobic metabolism of glycogen stores. The accompanying cytoplasmic acidification causes hyperactivation of plasma membrane NHE1 and the consequent accumulation of intracellular Na^+^. The Na^+^ overload reverses the mode of operation of Na^+^/Ca^2+^ exchange, driving excess Ca^2+^ into the cell. The resulting elevation in intracellular Ca^2+^ concentration, and subsequent activation of proteases, phospholipases, and formation of oxygen- and nitrogen-free radicals, precipitates a cascade of deleterious effects, including altered membrane excitability and contractility, generation of toxic free radicals, cellular hypertrophy, apoptosis, and necrosis—events that result in cell death [79, 80]. Activation of NHE also leads to the rapid normalization of pH_i_ during reperfusion after hypoxia ischemia [45, 54, 81, 82].

Removal of external Na^+^ or reducing the external pH attenuates the postanoxia alkalization [83]. When ATP levels fall to 35 % of control in isolated CA1 neurons during chemical anoxia, NHE activity is reduced by 44 %. Activation of NHE function, Na^+^ pump function and the Na^+^ driving for NHE are all dependent upon ATP energy levels, ATP being required for phosphorylation of the protein.

### NH Exchanger Blockade and Neuroprotection in Neonatal Models

Although the NHE activation is essential for the restoration of physiological pH_i_, hyperactivation of NHE1 in neurons, in response to the metabolic acidification associated with an ischemic–hypoxic insult [46, 84–87], disrupts the intracellular ion balance, causing intracellular Na^+^ and Ca^2+^ overload [88], which eventually leads to cell death. Experimental in vitro and in vivo studies have demonstrated neuroprotection with NHE inhibitors; these are summarized in Table 3.

**Table 3:** Preclinical studies—brain pH and NHE inhibitors in perinatal brain injury (based on [46, 84, 85, 88–90, 93, 101, 102, 107, 110, 111, 138, 145, 158–160])

Paper	Species	Model, study design	NHE inhibitor	Results
In vitro studies				
Vornov et al. [46]	Ex vivo Rodent 17-day rat fetuses 10–12 cell culture	–Neuronal tissue culture model of ischemia (18–19-day culture) from embryonic 17-day rat fetuses –20 min ischemia with metabolic inhibition (KCN + 2-DG) –Injury: LDH liberation	–Group 1: ischemic conditions vs. prolonged ischemia (30 min) –Group 2: incubation with NHE inhibitors at normal pH_e_ (dimethylamiloride and harmaline) slowed pH_i_ recovery	–Profound protective effects: ↓ pH_e_ during 1st hour recovery. →suggesting protective effects due to intracellular acidosis –1st demonstration of protective effects of blocking NHE in cerebral ischemia model (during recovery); worst injury if pH_i_ normalizes fast →acidosis protects: suppressing pH-sensitive mechanisms of injury or blocking Na entry (NHE)
Matsumoto et al. [88]	Ex vivo rodent 1-day rats Culture of cortical neurons	–Hypercapnia (5 % CO_2_) for 10–14 days, then cortical neurons cultured on glass-based dishes –Assess glutamate-induced neuronal death; neurons morphological change; Ca^2+^_i_ concentration and pH_i_	–Some given SM-20220 20 min preglutamate exposure or MK-801 (NMDA receptor antagonist)	–SM-20220: ↓ glutamate-induced neuronal death over 6 h, inhibited postglutamate exposure: acute cellular swelling, persistent ↑ [Ca^2+^]i and intracellular acidification →Neuroprotection: inhibit persistent ↑[Ca^2+^]i and acidification in excitotoxicity
Robertson et al. [89]	Ex vivo rodent 14- and 7-day models of rat pups Brain slices	–Progressive energy decline after HI insult in rat brain slice neonatal model; P^31^and H^1^ MRS 350 μm slice –7-day rat pups brain slices perfused in KHB: (1) at 37 °C; (2) at 32 °C, and 14-day slices perfused for 8 h in similar solutions and then NHE blocker	–14-day pups brain slices perfused for 8 h: (1) at 37 °C in KHB (2) at 32 °C in KHB (3) at 37 °C in HEPES buffer, (4) amiloride at 37 °C in HEPES	–No gestational age effect on energy decline between 7- and 14-day model –Brain slice model underwent secondary energy failure At 5 h: alkaline pH_i_, ↓ PCr/Pi and ↑ Lac/NAA, and ↓ NTP/PME, at 37°C –Changes delayed with hypothermia (32^o^ C) or amiloride (pH_i_ acidified and preserved NTP/PME, at baseline and at 5 h)
Kersh et al. [85]	Ex vivo rodent 3–15-day rat both sex Brain slices	–Hypercapnia (15 % CO_2_) –NH_4_Cl-induced acidification in brainstem neurons from chemosensitive regions of neonatal rats (brainstem slices from RTNn, NTSn, and LCn)	–Control (DMSO-vehicle) –Amiloride –HOE 642 –S1611 –EIPA	–pH_i_ recovery mediated by different pH-regulating transporters in neurons from different chemosensitive regions (NHE1 in RTNn; NHE1 and 3 in NTSn; NBC in LCn) –Recovery suppressed by hypercapnia in all neurons (maintained acidic pH)
Liu et al. [90]	Ex vivo rodent 1–3-day neonatal mice Glial cultures	–Isolation of mixed primary glial cultures in mice –Activation of microglia after lipopolysaccharide or oxygen and glucose deprivation and reoxygenation	–Group 1: untreated –Group 2: HOE 642	–HOE 642 abolished pH_i_ regulation in microglia basal conditions –Activation of microglia accelerated pH_i_ regulation (↑ pH_i_, ↑ Na^+^_i_ and Ca^2+^i, and production of superoxide anion (SOA) and cytokines (CK)) –HOE 642 abolished pH_i_ regulation, ↓ production SOA, CK and _i_NOS –Hypothesis: NHE1 to maintain microglial pH_i_ homeostasis (NADPH oxidase and “respiratory” burst)
In vivo studies				
Ferimer et al. [158]	Rodent 13 Wistar rats	Cardiac arrest (KCl) in rats followed by resuscitation 7 min later in untreated vs. MIA	MIA Controls (untreated)	–MIA delays normalization of brain pH_i_ after cardiac arrest in rats –MIA: ↓ cardiac pH in rats postarrest +15 min reperfusion –MIA doesn’t change pH_i_ from nonischemic value.
Phillis et al. [107]	Rodent 21 Sprague–Dawley rats	–Ischemia: 20 min occlusion CA (group 3 30 min), with EEG (flat). Then 40 min reperfusion –Cortical superfusate (bilaterally every 10 min): free fatty acids (FFA), lactate, and glucose levels	–Group 1 (*n* = 9): aCSF (control) –Group 2 (*n* = 6): EIPA topical (cortex) 35 min pre- and during ischemia –Group 3 (*n* = 6): 30 min ischemia	–NHE inhibition prevented activation phospholipases (suppress ↑ FFA during reperfusion) –EIPA: lactate levels significantly lower by end of experiment
Pilitsis et al. [110]	Rodent 24 Sprague–Dawley rats	–Cerebral ischaemia (20 min CA occlusion) –Measurement of phospholipase activation by efflux of FFA in the ischemic/reperfused rat cerebral cortex	–Group 1: SM-20220 topical (cortex) pre- and during ischemia (*n* = 13) –Group 2: control (ischemia) (*n* = 11)	–↓significantly ischemia-evoked efflux of FFAs: importance NHEs in eliciting FFA efflux –Inhibition may be essential for neuroprotection in ischemia–reperfusion injury
Kendall et al. [93]	Rodent 47 mice 7-day (adult C57/Bl6 female and males bred in-house)	–HI: 2 h left CA occlusion followed by moderate (30 min) or severe (1 h) hypoxia (8 % O_2_) –Outcome at 48 h: viable tissue in injured hemisphere (severe HI) or injury score and TUNEL stain (moderate)	–Group 1: MIA intraperitoneal –Group 2: 0.9 % saline equivalent volume Given 8 hourly starting 30 min before HI	–MIA neuroprotective when commenced before HI (no weight difference) –Severe insult: significant neuroprotective (↑forebrain tissue survival) –Moderate insult: ↓ damage hippocampus –MIA ↓ neutrophil count and hence brain swelling after HI
Rocha et al. [159]	Rodent 3–4-month mice male Swiss-Webster	–Metabolic stress and dopaminergic damage in mice caused by malonate (mitochondrial inhibitor) –Dialysate levels of DA and metabolites baseline (1 h prior to drug delivery) and afterwards, every 20 min	–Group 1: HOE-642 dialized intracerebral (striatum) 20-min periods, separated by drug washout ≥1 h –Group 2: EIPA –Group 3: control (only malonate)	–HOE-642 pretreatment: ↓malonate-induced DA overflow and ↓ striatal DA content, without ↓ intensity metabolic stress or subsequent DAergic axonal damage –Absence NHE1 on nigrostriatal DAergic neurons suggests HOE-642 effects on striatal DA overflow via NHE1 on other cell types or via multiple NHE isoforms
Hwang et al. [84]	Rodent 6 m Mongolian gerbils	–HI by 5 min bilateral occlusion common CA –Assess delayed neuronal death and immunohistochemistry for NHE1 (at 30 min, 3 h, 12 h and 1, 2, 3, 4, and 5 days following surgery) –Locomotor activity monitored for 10 days post-HI	–Group 1: normal (sham: same surgical procedure but NO ischemia) –Group 2: vehicle (saline given) –Group 3: EIPA OD for 3–9 days after ischemic sugery, starting 30 min postischemic surgery	–↑NHE protein level in CA1 region from 2 days post-HI; activation NHE1 in CA1 glial cells from 2 to 3 days post-HI; in CA1 pyramidal neurons and glial cells(astrocytes) from 4 days –EIPA potently protected CA1 pyramidal neurons from ischemic injury, and ↓ activation of astrocytes and microglia in ischemic CA1 region –Hypothesis: role of NHE1 in delayed death NHE inhibitors protect neurons from ischemic damage
Shi et al. [101]	Rodent 136 mice –NHE1^+^/^−^ heterozygous mice –Wild-type mice SV129/Black Swiss –NHE1^+^/^−^ and ^+^/^+^ litter mate males	–Transient focal cerebral ischaemia and reperfusion (I/R) by 60 min occlusion left MCA –Activated microglial cells identified by expression of 2 microglial marker proteins (CD11b and Iba1) and by transformation of morphology	–Group 1: vehicle control (equivalent volume of saline intraperitoneal) –Group 2: HOE 642 intraperitoneal at 30 min prior to the onset of reperfusion, and then daily up to 1–7 days during reperfusion	–Immediate ↑ microglial activation ipsilateral to ischemia in NHE1^+^/^+^ brains at 1 h I/1 h R (gradually ↓ during 6–24 h) Sharp ↑ microglial activation peri-infarct and ↑ proinflammatory CK 3 days after I/R –HOE 642 or NHE1^+^/^−^ mice: less microglia activation, lNADPH oxidase activation, ↓ proinflammatory response at 3–7 days post-I/R Blocking NHE1 significantly ↓ microglial phagocytosis in vitro –↑↑ NHE1 protein expression in activated microglia and astrocytes NHE1 inhibition ↓ microglial proinflammatory activation following I/R
Ferrazzano et al. [160]	Rodent 44 wild-type controls (NHE1^+^/^−^), NHE1 genetic knockdown mice (NHE1^+^/^−^)	–Transient focal cerebral ischemia by 30–60 min occlusion of left MCA induced in wild-type controls (NHE1^+^/^+^), NHE1 genetic knockdown mice (NHE1^+^/^−^), and NHE1^+^/^+^ mice treated with HOE-642 –Brain MRI (diffusion DWI and T2 weighted)	Randomised to: –Group 1: HOE 642 30 min pre- or 1 h postreperfusion intraperitoneally. Then at 24 and 48 h after reperfusion –Group 2: control (saline as vehicle)	–Significant protection in NHE1^+^/^−^ mice ↓injury in DWI 1 h postreperfusion in NHE1^+^/^−^; and smaller infarct in T2 at 72 h vs NHE1^+^/^+^mice –HOE642 prereperfusion or during early reperfusion: ↓ ischemic damage (remains protective given during early reperfusion!) →Therapeutic potential for inhibition NHE1 in cerebral ischemia
Cengiz et al. [102]	Rodent 9 days 46 C57BL/6J mice	–30 min unilateral ligation of the left common CA, plus exposure to hypoxia (8 % O_2_ for 55 min) –Assessment of morphology, neurodegenerationand motor and spatial learning abilities at 4–8 weeks of age after HI	Randomised to: –Group 1 (*n* = 13): HOE 642 intraperitoneal: 5 min pre-HI, 24 and 48 h post –Group 2 (*n* = 10): control (saline) pre/posttreatment –Group 3 (*n* = 13): HOE-642 posttreatment (10 min, 24 and 48 h post-HI) –Group 4 (*n* = 10): control (saline) post	Inhibition of NHE1: neuroprotective in neonatal HI brain injury –Control brains 72 h post-HI: neurodegeneration in several areas brain; NHE1 upregulated in specific astrocytes; and motor-learning deficit seen at 4 weeks age –HOE 642: better preserved morphologic hippocampal structures; less neurodegeneration in acute stage HI; and improved striatum-dependent motor and spatial learning at 8 weeks of age after HI →NHE1-mediated disruption of ionic homeostasis contributes to striatal and CA1 pyramidal neuronal injury after neonatal HI
Helmy et al. [138]	Rodent 6 days 159 Male Wistar rat pups	–60 min of asphyxia by hypoxia 9 %, or hypercapnia 20 %, or both combined. Then normal restoration of room air or graded re-establishment of normocapnia (half CO_2_ levels every 30 min) –Monitoring with EEG recording and pH-sensitive microelectrodes	Some in each group: MIA intraperitoneally 30 min preasphyxia –Group 1 (60 min hypoxia 9 % then 21 %) –Group 2 (60 min hypercapnia 20 %) –Group 3 (asphyxia: CO_2_ 20 % + O_2_ 9 %) –Group 4 (asphyxia like group 2 and then graded re-establishment of normocapnia) –Group 5: controls (room air only)	–Recovery from asphyxia followed by large seizure burden and ↑ brain pH –Graded restoration of normocapnia after asphyxia strongly suppresses alkaline shift in brain pH and seizure burden –MIA pre-insult: virtually blocked seizures
Helmy et al. [145]	Rodent 6–7 days Male Wistar rat pups	–60 min of asphyxia by hypoxia 9 % and hypercapnia 20 %. Then normal restoration or graded re-establishment of normocapnia (half CO_2_ levels every 30 min) –Monitoring with EEG recording, pH-sensitive microelectrodes and histology	5 pups in each group: MIA intraperitoneally 30 min pre-HI A few: amiloride intraperitoneally 30 min preasphyxia –Group 1 (asphyxia CO_2_ 20 % + O_2_ 9 %, then room air) –Group 2 (asphyxia like group 1 and then graded restoration normocapnia)	–Neocortical neurons in vivo: biphasic pH changes acid–alkaline response –Graded restoration normocapnia: strongly suppress alkaline overshoot –Parallel ↑ pH_e_ and pH_i_ post-HI: net loss acid equivalents from brain tissue not attributable to BBB disruption (lack of ↑Na fluorescein extravasation into brain and EEG characteristics of BBB) –MIA: abolition net efflux acid equivalents from brain, and suppression seizure (sz) activity –Post-asphyxia sz: due to brain alkalosis (NHE-dependent net extrusion acid across BBB) –BBB-mediated pH regulation: new approach prevention and therapy neonatal sz
Robertson et al. [111]	Piglet 18 white male <24 h old	–Transient global cerebral HI (bilateral occlusion common CA) ^31^P and ^1^H MRS before, during and up to 48 h after HI. Tissue injury at 48 h	Randomized to: –Saline placebo –iv MIA 10 min post-HI and 8 hourly	–MIA starting 10 min after severe HI: neuroprotection: ↓ brain Lac/NAA, cell death and microglial activation

Abbreviations: *NHE* Na^+^/H^+^ exchanger, *NHE1* isoform 1 of NHE, *NCX1* Na^+^/Ca^2+^ exchanger-1, *NBC* Na- and HCO_3_-dependent transporter, *KCN* potassium cyanide, *HI* hypoxia-ischemia, *CA* carotid arteries, *BBB* blood–brain barrier, *MIA N*-methyl-isobutyl-amiloride (inhibitor of NHE), *EIPA N*-(*N*-ethyl-*N*-isopropyl)-amiloride (highly potent derivative of amiloride for the nonselective inhibition of the NHE system in various cell types), *SM-20220 N*-(aminoiminomethyl)-1-methyl-1*H*-indole-2-carboxamide methanesulfonate (a highly selective and specific NHE1 inhibitor, 50 times more potent than EIPA), *HOE-642* cariporide mesilate or 4-isopropyl-3-methylsulfonylbenzoyl-guanidine methanesulfonate (a selective NHE1 inhibitor), *S1611* (a selective NHE3 inhibitor), *Harmaline* (a non-amiloride NHE5 inhibitor), *NTP/PME* nucleotide triphosphate/phosphomonoester, *Pi* inorganic phosphate, *PCr* phosphocreatine, *Lac/NAA* lactate/NAA ratio, *RTNn* retrotrapezoid nucleus neurons, *NTSn* nucleus tractus solitarii neurons, *LCn* locus coeruleus neurons

There are two major classes of pharmacological NHE inhibitors. One class includes amiloride and its derivatives by double substitution of the nitrogen of the 5-amino group: dimethylamiloride (DMA), *N*-(*N*-ethyl-*N*-isopropyl)-amiloride (EIPA), *N*-methyl-isobutyl-amiloride (MIA), and 5-(*N*,*N*-hexamethylene)-amiloride. They are nonselective inhibitors of the NHE system in various cell types, and EIPA is highly potent. Another class of inhibitors includes the derivatives of the benzylguanidines such as HOE 694, HOE 642 (cariporide mesilate or 4-isopropyl-3-methylsulfonylbenzoyl-guanidine methanesulfonate), eniporide, and BIIB-513, which selectively inhibited NHE1. The replacement of the pyrazine ring of amiloride by a pyridine ring or by a phenyl increased the potency and the NHE selectivity. In the last two decades, several bicyclic guanidines were prepared: zoniporide, MS-31038, SM-20220, SM-20550, SMP-300, KBR9032, BMS-284640, T-162559, TY-12533, S-3226, or SL-591227 [52]. Of these, HOE 642 is extremely potent, being 105 times more specific for NHE1 vs. NHE3 [78], and zoniporide is very potent too and 150-fold selective for NHE1 vs. the other isoforms. S1611 and S3226 are two selective NHE3 inhibitors. Harmaline is a nonamiloride NHE5 inhibitor. The inhibitory potency of NHE inhibitors towards the different NHE isoforms is described in detail in a review by Masereel et al., [52].

During in vitro cerebral ischemia, NHE inhibitors (DMA, harmaline [46], SM-20220 [88], amiloride [89], and HOE-642 [85, 87, 90]) had a protective effect, in terms of delayed pH_i_ normalization both in neuronal cells and in astrocytes [46, 89–92], inhibition of microglia [90], and less intracellular calcium accumulation [87, 88]. This anoxia-induced alkalization was also ameliorated in NHE1−/− CA1 neurons. In parallel, cell death was reduced in wild-type neurons treated with HOE 642 or in NHE1−/− neurons in cultured mouse cortical neurons undergoing 3 h oxygen–glucose deprivation (OGD) and 21 h reoxygenation (from ∼70 % to 40–50 %) [87].

Vornov et al. [46], described that pH_i_ decreased by 0.2 pH units, but then recovered when inhibition was removed in rat cortical neuronal cultures undergoing metabolic inhibition for 20 min. NHE1 inhibitors, dimethylamiloride or harmaline, significantly reduced the postinhibition pH_i_ recovery, showing less brain injury in these compared to those with a fast pH_i_ normalization.

As mentioned above, NHE1 activity also affects cellular Na^+^ levels. A small but significant (∼2-fold) increase in neuronal intracellular concentration of Na^+^ ([Na^+^]i) results after 2 h OGD, reaching to ∼7-fold increase during 1 h reoxygenation. HOE 642 attenuated the rise in [Na^+^]i following OGD. NHE1−/− neurons did not exhibit significant increase in [Na^+^]i. This supports that NHE1 activity is elevated upon reoxygenation [83, 86]. Three hours of OGD and 21 h reoxygenation lead to cell death in ∼70 % of the NHE1+/+ neurons. However, cell death is significantly reduced in wild-type neurons treated with HOE 642 or in NHE1−/− neurons [87].

In vivo studies mainly in rodents have found that NHE1 inhibitors reduce brain injury in hypoxia–ischemia models [93] and decrease infarct size in a focal ischemia model [87]. In NHE1 heterozygous mice, there was a significant decrease in infarct size of around 30 % compared with normal NHE+/+ mice following middle cerebral artery occlusion (MCAO) [87], similar to those NHE+/+ mice treated with HOE 642. It implies that the disruption of Na^+^ and Ca^2+^ homeostasis contributes to ischemic neuronal damage. These studies firmly demonstrate the dominant role of NHE1 among other NHE isoforms in cerebral ischemic brain damage.

Other studies show neuroprotection in ischemic models. SM-20220 (*N*-(aminoiminomethyl)-1-methyl-1H-indole-2-carboxamide methanesulfonate), a highly selective NHE1 inhibitor, given intravenously 1 h after MCAO significantly reduced the extent of cerebral edema and Na^+^ content after 2 h ischemia and 4 h reperfusion and infarct volume after 22 h reperfusion [94]. SM-20220 decreased infarct size in both transient and permanent MCAO models. The reduction of infarct size improved when the treatment is delayed for 5, 30, or 60 min after the onset of ischemia [94]. SM-20220 led to ∼50 % decrease in infarct size at 72 h reperfusion. Other groups have shown similar reductions in infarct volume and edema with other NHE blockers [95–97].

Some NHE blockers, specifically SM-20220 [98] and MIA [93], have been found to reduce the number of neutrophils in the ischemic hemisphere. SM-20220 attenuates leukocyte adhesion and migration in the mesenteric artery [99] and improves endothelial dysfunction [100]. NHE1 blockers (EIPA and HOE 642) [84, 90, 101, 102] reduced microglial activation posthypoxia ischemia and hence the microglial phagocytosis and astrocytosis in CA1 region. Cengiz et al. [102] found that HOE 642 given either prehypoxia–ischemia or starting 10 min after the insult reduced neurodegeneration in the acute stage in hippocampus, striatum, and ipsitaleral thalamus, and improved the striatum-dependent motor and spatial learning skills at 8 weeks.

NHE1 inhibitors are neuroprotective in astrocytes in a similar way to neurons. The intracellular Na^+^ overload is substantially mediated through activation of the ERK1/2 pathways [92]. In a more severe model of in vitro ischemia, cultured astrocytes were superfused with a solution, which mimics the ionic composition of the ischemic extracellular space (a hypoxic, acidic, ion shifted ringers (HAIR) solutions at 37 °C) [103, 104]. Exposure to HAIR caused a rapid decline in astrocyte pH_i_. This is consistent with the finding that NHE1 activity is inhibited by low extracellular pH (pH_e_) [105]. When exposed to normal buffer again, astrocytes rapidly alkalized and a significant rebound of pH_i_ over the baseline took place [104]. Bondarenko et al. [104] have reported that HAIR and reoxygenation leads to ∼40 % cell death in astrocytes, which is significantly reduced in the presence of NHE blockers such as EIPA or HOE 694 during HAIR and reoxygenation, or during reoxygenation alone.

NHE inhibitors have also been shown to preserve neurological function after ischemic injury. In a model of ischemia and hypothermia in piglets, Castella et al. [106] observe a rapid neurological recovery in subjects receiving HOE 642 just at the onset of cooling. Several studies [84, 107] demonstrate that EIPA not only protects gerbil hippocampal neurons from ischemic injury but also reduces the magnitude of the ischemia-induced locomotor hyperactivity at both 24 h and 6 days after reperfusion. Ischemic injury to the gerbil forebrain produces an increase in locomotor activity, which is related to the degree of pyramidal neuronal damage in the CA1 region of the hippocampus [84, 108]. In another Mongolian gerbil model, the recovery time of consciousness significantly improved (lower neurological scores at 2 h postreperfusion) following a 30 min transient global cerebral ischemia in those treated with SM-20220, compared to the vehicle group [109]. This improvement in neurological deficit persisted until 24 h postreperfusion.

In summary, NHE inhibitors (or genetically modified NHE1−/−) are neuroprotective in animal models. They reduce the alkalinization of the cell (inhibition of phospholipases and kinases [107, 110] and delayed secondary energy failure [89]), and decrease the calcium and sodium intracellular overload, reducing cell edema and cell damage. They suppress microglial activation, especially in hippocampal regions (pyramidal neurons of the CA1 area in rodents) [84] and in striatum and ipsilateral thalamus in a focal ischemia model [102]. Improved functional outcome also follows NHE inhibition [102].

### NHE Inhibition Following Transient Hypoxia–Ischemia in the Piglet Model of Perinatal Asphyxia

Robertson et al. [111] used the piglet model of perinatal asphyxia to study the effect of NHE1 inhibition with MIA given 10 min after the end of hypoxia–ischemia and eight hourly thereafter. MIA improved cerebral energy metabolism in the thalamic area in the 48 h after hypoxia–ischemia on MRS biomarkers and reduced cell death and microglial activation in some brain areas. The use of clinically relevant MRS biomarkers and reduced cell death in a large animal model of perinatal asphyxia is an important step towards the clinical translation of NHE inhibitors. It is important to assess whether NHE blockade would augment hypothermic neuroprotection in this devastating disease.

## Brain pH_i_ and Seizures

### Relation Between Brain pH_i_ and Seizures

Neonatal seizures are the most common manifestation of a neurological disorder in the newborn period. Neonatal encephalopathy is the most frequent cause of seizures at term. In some studies, the duration of electrographic seizures in newborn babies is associated with worse MRI appearances [112] and poor neurodevelopmental outcome [113]. Newborn babies with neonatal encephalopathy as a cause of seizures tend to have a higher seizure burden than those with a stroke [113]. Increased morbidity and mortality, brain injury, and poor neurodevelopmental outcome have been associated with a higher seizure burden [113–120]. Therefore, addressing neonatal seizures is a priority, but current drugs are largely ineffective and are not free of serious side effects on the neonatal brain [121–123].

Therapeutic hypothermia not only improves outcomes of babies with neonatal encephalopathy by reducing the rate of death and disability at 18 months of age, but it also appears to reduce seizure burden [124]. Cooling appears either to act directly to reduce seizure burden or to augment the action of conventional anticonvulsants. Xenon gas, which is currently being used in clinical phase II trials to augment hypothermic neuroprotection in babies, appears to reduce seizure burden when administered with cooling [125]. The data are consistent with those from preclinical models [124].

There is however still an unresolved controversy whether seizures worsen outcome by themselves or whether they are associated to those with a more severe degree of encephalopathy, hence the higher mortality and morbidity [126, 127]. The molecular and cellular mechanisms underlying birth-asphyxia seizures are unknown, but understanding the seizure-triggering mechanisms plays a key role in the design of novel therapeutic strategies.

A profound acidosis (blood pH 7.00 or lower) at the time of birth is an essential criterion in the diagnosis of perinatal asphyxia [128, 129]. However, postasphyxia seizures do not coincide with the maximal blood acidosis but are typically first observed during the recovery period after a delay that usually ranges in human babies from 2 to 16 h [130]. A wide range of observations have shown that changes in extra- and intracellular pH exert a strong modulatory effect on brain excitability under normal and pathophysiological conditions, whereby an alkalosis enhances excitability while an acidosis has an opposite effect [131–138]. The immature brain appears to be particularly sensitive to changes in pH_i_. Recent experiments have shown that changes of 0.05 pH units on neonatal hippocampal slices have a profound effect on endogenous network activity [137].

In a rat pup model of neonatal asphyxia, brain alkalosis after recovery from asphyxia played a key role in the triggering of seizures [138]. Brain pH was measured with intracortical ion sensitive microelectrodes, and EEG was recorded from parietal cortex in freely moving rat pups with simultaneous video recording [138]. There was a robust correlation between postasphyxic brain pH changes and seizure burden; that is, the more alkaline the brain pH after asphyxia, the higher the seizure burden. The alkaline brain pH overshoot after asphyxia reached values of up to 0.40 pH units above normal brain pH; these are similar finding to previous studies in rats [93], piglet [3, 111], and human newborn [4, 5]. Some pups received MIA (the NHE inhibitor) intraperitoneally 30 min before asphyxia; in those rat pups pretreated with MIA, seizure burden was reduced. These data add weight to the evidence that acidosis suppresses neuronal excitability, whereas alkalosis does the opposite [133, 139–141]. Taking this further, there is accumulating evidence that brain pH_i_ plays a key role in modulating neuronal survival after injury [131, 142, 143].

In another study in neonatal rat pups by the same group, a rise in brain pH was accompanied by seizure activity following injection of sodium bicarbonate [144]. The authors suggested that a “graded restoration of normacapnia” should be recommended for resuscitation of neonatal encephalopathy as this may reduce seizures and improve outcome [144].

The same group explored mechanisms of postasphyxia brain alkalosis [145] in the rat pup exposed to one hour of asphyxia (simultaneous hypoxia and hypercapnia). They observed that the postasphyxia brain alkalosis was generated in the absence of a rise of blood pH. The increase in both extracellular and intracellular pH recorded in the brain implied an enhanced net loss of acid equivalents from brain tissue across the BBB. BBB disruption itself was excluded by the absence of increased sodium fluorescein extravasation into the brain and by electrophysiological characteristics of the BBB [145]. The net efflux of acid equivalents from the brain across the BBB was abolished by MIA, suppressing the postasphyxia rebound alkalosis of the brain extracellular pH without having any significant effect on the weight of the pups. Remarkably, MIA also abolished postasphyxia seizures (reduced seizure burden by 88 %) when applied preinsult in rat pups [138, 145] and ameliorated brain injury when applied before hypoxia–ischemia in neonatal mice [93]. These findings support the conclusion that activation of NHE in the BBB leads to brain alkalosis and consequent seizures.

Therapeutic hypothermia is now standard care for infants in the developed world with moderate to severe neonatal encephalopathy [146]. It is unknown if NHE inhibition combined with therapeutic hypothermia might augment hypothermic neuroprotection after perinatal asphyxia. Some in vitro studies suggest that moderate hypothermia (reducing temperature from 37 to 20 °C) itself increases NHE activity [147, 148], while others suggest that hypothermia produces a partial inhibition of NHE activity [149]. The combined effects of an NHE inhibitor with hypothermia could significantly augment hypothermic neuroprotection especially as they act on separate pathways. Importantly, studies suggest that the potency of NHE inhibition under hypothermic conditions is not influenced by a change in temperature [149] and NHE inhibition was beneficial in a perfused heart model even after hypothermic ischemia [150]. Preclinical large animal studies of combined therapy with cooling are high priority.

## Summary

Perinatal hypoxic–ischemic brain injury in term babies is still a significant problem throughout the world. Therapeutic hypothermia has improved outcome, especially of those with a moderate insult. Unfortunately, around 40 % of infants treated with therapeutic hypothermia still have adverse outcomes. Experimental data suggest that the addition of another agent to cooling may enhance overall protection either additively or synergistically. Experimental models have demonstrated the central role of NHE in maintaining brain pH_i_ and ion homeostasis, and in microglial activation in some areas of the brain. NHE inhibitors are a promising neuroprotective agent in animal models, improving brain energy metabolism in in vivo MRS, reducing cell death, seizure burden, and brain injury with improvement of functional outcome. Preclinical studies are urgently needed to assess possible augmentation of hypothermic neuroprotection with NHE inhibition.
